# Study on SR-Crossbar RF MEMS Switch Matrix Port Configuration Scheme with Optimized Consistency

**DOI:** 10.3390/s24103099

**Published:** 2024-05-13

**Authors:** Weiwei Zhou, Weixing Sheng, Binyun Yan

**Affiliations:** School of Electronic and Optical Engineering, Nanjing 210094, China; willwill@njust.edu.cn (W.Z.); yanby@njust.edu.cn (B.Y.)

**Keywords:** RF MEMS, switch matrix, topology, port configuration, consistency

## Abstract

The performance consistency of an RF MEMS switch matrix is a crucial metric that directly impacts its operational lifespan. An improved crossbar-based RF MEMS switch matrix topology, SR-Crossbar, was investigated in this article. An optimized port configuration scheme was proposed for the RF MEMS switch matrix. Both the utilization probability of individual switch nodes and the path lengths in the switch matrix achieve their best consistency simultaneously under the proposed port configuration scheme. One significant advantage of this scheme lies in that it only adjusts the positions of the input and output ports, with the topology and individual switch nodes kept unchanged. This grants it a high level of generality and feasibility and also introduces an additional degree of freedom for optimizations. In this article, a universal utilization probability function of single nodes was constructed and an optimization objective function for the SR-Crossbar RF MEMS switch matrix was formulated, which provide a convenient approach to directly solving the optimized port configuration scheme for practical applications. Simulations to demonstrate the optimized dynamic and static consistencies were conducted. For an 8 × 8 SR-Crossbar switch matrix, the standard deviations of contact resistances of 128 units and losses of all 64 paths decreased from 1.00 and 0.42 to 0.51 and 0.23, respectively. These results aligned closely with theoretical calculations derived from the proposed model.

## 1. Introduction

As RF systems continue to evolve towards high integration, multifunctionality, and reconfigurability, RF switch matrices play an increasingly vital role in them. This is especially true in compact systems, where RF switch matrices must meet the demands of high performance and good reliability while maintaining a compact size. Furthermore, spaceborne applications impose strict requirements on the size and weight of devices. While traditional mechanical coaxial switch matrices boast superior RF performance, their larger size and weight are not suitable for highly integrated spaceborne environments. Therefore, there is a demand for designing RF switch matrices that are compact, lightweight, and with good performance. RF MEMS switches, characterized by low power consumption, low loss, high linearity, high isolation, and small size, are thus becoming the ideal solution in such systems [1].

However, the short lifespan of RF MEMS switch units significantly hinders their further development and application [2]. This issue also applies to matrices composed of RF MEMS switch units. Additionally, due to the presence of multiple switch units in RF paths in the matrix, there is an accumulative effect on path loss, amplifying the performance discrepancies of the switch units within the paths. Consequently, using the switch matrix may lead to a situation where, although the degradation of individual switch unit performance is within an acceptable range, the accumulative losses in the matrix paths result in a substantial increase in overall performance degradation. The disparities in performance between paths are also greatly amplified, potentially exceeding acceptable limits, which ultimately leads to the failure of switch matrices. These issues present a new challenge in the design of RF switch matrices.

An RF switch matrix is designed to route RF signals by selectively connecting different RF paths. Each RF path contains multiple RF switches, and there are variations in both the length and structure of each path. For an RF switch matrix, it is desirable not only to maintain good performance at a specific moment but also to ensure that the performance and its consistency do not undergo significant changes during matrix usage. In other words, the RF performance consistency of the switch matrix manifests in two different aspects:

1. Static consistency: This is determined by the fixed, static design, such as the design of individual switch units and the topology of the matrix. It primarily manifests in the uniformity of path lengths and structures. This type of consistency does not change during the usage of the switch matrix. In this article, we define it as static consistency.
2. Dynamic consistency: This is determined by the performance degradation during the usage of the switch matrix. It mainly manifests in the uniformity of performance degradation of switch units, which is related to the frequency of usage for switch units. In this article, we define it as dynamic consistency.

For non-blocking RF MEMS switch matrices, the lengths and positions of signal paths vary depending on connections between different input and output ports. Compared to smaller switch devices, these matrices tend to contain more switch units, and the path lengths and number of switch units in different switching scenarios vary substantially.

Therefore, for various non-blocking RF MEMS switch matrices, there exist phenomena of uneven path lengths and the uneven utilization of switch units within the matrix, which will cause performance degradation.

Possessing excellent static and dynamic consistency ensures that the RF MEMS switch matrix maintains outstanding performance throughout its operational life, thus prolonging its lifespan.

Previous research efforts have primarily focused on improving the static consistency of switch matrices. Enhancing static consistency can be achieved through methods such as increasing the reliability of switch units and optimizing the topology of switch matrices. For switch units, static consistency is directly related to unit reliability. Reliability problems of capacitive switches are often related to dielectric charging [2]. Article [2] conducted extensive research on the dielectric charging of capacitive RF MEMS switches, which provided a better understanding of the failure mechanism. For metal-contact switches, the reliability problem mainly lies in contact degradation and the increase in contact resistance. Hence, optimizing the uniformity of utilization probabilities of switch units in a matrix will be an effective approach to enhance the dynamic consistency, since less contact degradation and contact resistance variations among switch units can be expected. Other optimization approaches include material selection, restoring the mechanism design, electrical protection mechanism implementation, fluid filling, and so on [3,4,5,6]. In paper [7], an analysis of contact materials and contact damage mechanisms for RF MEMS switches was conducted to improve the reliability of switch units. The mechanically coupled structure designed in paper [8] applies additional restoring force to the switch, resulting in enhanced anti-adhesion capability compared to conventional switches. Additionally, applying protection to switch contacts is an effective measure. In paper [9], a pre-contact mechanism was designed such that protective contacts absorb the initial contact force, which reduces degradation on the main contact and enhances the thermal switching reliability of switch units.

Regarding topology, common structures include various variants of Benes and crossbar structures. Paper [10] designed an RF MEMS switch matrix with a Benes structure, which exhibited superior path uniformity and modular design capability. However, this switch matrix structure suffers from a lack of compactness. Crossbar structures are advantageous for large-scale matrices due to their compactness and ease of expansion. Paper [11] designed and fabricated a switch matrix with a crossbar structure, which was compact and easy to expand. However, a major drawback of the crossbar structure is its subpar static consistency among different RF paths, particularly evident at higher orders of switch matrices [12]. Addressing this, an L-shaped matrix structure was proposed in paper [13]. This structure retains the compactness and expandability advantages of a crossbar but with less favorable port layouts for chip fabrication and integration.

Combining the advantages of the aforementioned switch matrix topologies, paper [14] designed the SR-Crossbar topology. This topology significantly reduces the number of switch units used in the switch matrix, thereby enhancing the overall reliability and RF performance of the switch matrix. Additionally, due to the reduction in switch units in the paths, the static consistency of each path is also improved.

These studies have conducted extensive research from various aspects, greatly enhancing the reliability of RF MEMS switch units. Additionally, by employing different designs of switch matrix topologies, they have improved the overall matrix performance. While these studies have evidently improved the matrix static consistency, research on the dynamic consistency of RF MEMS switch matrices is relatively rare. Given that dynamic consistency optimization can be conducted without modifying switch units or matrix topology, thus keeping the optimized static consistency, it can significantly enhance the overall consistency of the switch matrix.

This article begins by focusing on dynamic consistency to optimize the overall consistency in RF MEMS switch matrices with the SR-Crossbar topology. While maintaining switch units and matrix topology unchanged, the optimization to improve the consistency of a switch matrix is achieved only by adjusting the input and output port configuration scheme.

## 2. Modelling and Analysis

**Figure 1:**
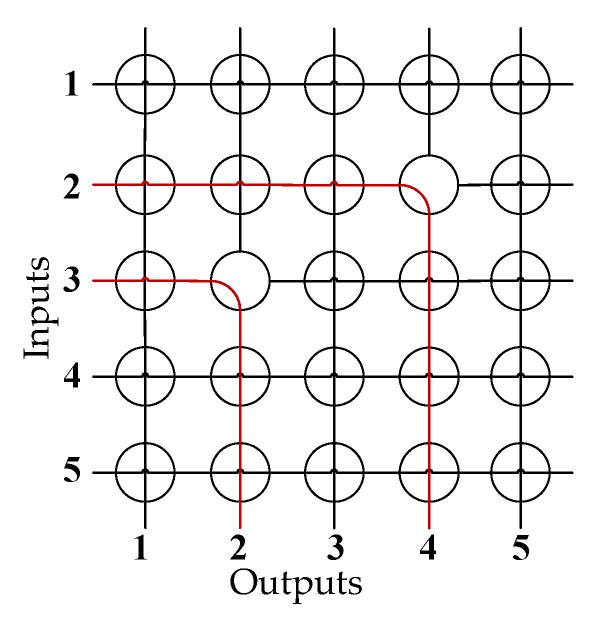
A crossbar switch matrix with input 2 to output 4 and input 3 to output 2 connected.

In RF MEMS switch matrices, crossbar topology is simple and easily scalable, which makes it one of the most commonly used network topologies. For switch matrices based on the crossbar topology, input and output ports are typically distributed along two adjacent sides of the matrix. Signals entering through input ports into corresponding rows/columns are routed via turning nodes to output columns/rows and then exit through output ports. These nodes, composed of switch units, can work at different states to route signals in various directions. From this working principle, it can be inferred that in scenarios where inputs and outputs are randomly interconnected without being blocked, the units near the input and output ports are more likely to be utilized, while those farther away have a lower probability of utilization. Over time, this leads to more significant performance degradation in units near the ports, which reduces the dynamic consistency of the entire switch matrix. Figure 1 shows a crossbar switch matrix.

Therefore, the key to optimizing the dynamic consistency of the switch matrix lies in ensuring that the probability of each switch unit being utilized is as uniform as possible in the matrix. Meanwhile, by just modifying the configuration of the input and output ports while keeping the switch unit and matrix topology unchanged to optimize overall matrix consistency, this approach offers an additional degree of freedom to improve the overall lifespan of the switch matrices. Thus, this port configuration optimization approach is broadly applicable to crossbar-based switch matrices, independent of the switch unit types composing the matrix, which makes the approach highly versatile.

The SR-Crossbar structure proposed in paper [14] improves the overall reliability and performance of RF switch matrices by reducing the number of switch units in a crossbar matrix. This article builds upon this foundation to further research optimizing the input and output port configurations to enhance dynamic consistency.

As analyzed above, for the SR-Crossbar switch matrix with random non-blocking interconnections between the inputs and outputs, the switch units near the inputs and outputs generally have a higher probability of being utilized, while the switch units far from the ports have a lower probability of being utilized. Therefore, this leads to more severe performance degradation in the units near the ports, creating significant inconsistencies in the performance of the entire switch matrix. Thus, accurately establishing a utilization probability model for the matrix nodes is key to analyzing and optimizing the port configuration schemes.

In the following text, we will provide a detailed analysis of the probability of each node being utilized in an SR-Crossbar switch matrix with random non-blocking interconnections between any input and output ports.

Assuming an SR-Crossbar RF MEMS switch matrix with M rows and N columns, we conducted an analysis on the switch matrix node located at (m,n). Here, M, N, m, and n satisfy the following conditions: M, N∈Z, M, N≥2, m=1,2,3,⋯,M, and n=1,2,3,⋯,N.

For any RF MEMS switch matrix based on a crossbar topology, there exists a critical node in each established path between the input and output ports. This node directs a signal from the input row/column to output column/row. In this article, we refer to this node as the “turning node”, denoted as TN. For example, in Figure 1, nodes (3,2) and (2,4) are both turning nodes (TNs). 

As per the non-blocking characteristic of the crossbar matrix topology, at any given time, there is only one working TN in the same row and same column. Additionally, given that the input and output ports are fixed in practical applications, a TN uniquely corresponds to an RF signal path, with other nodes in the path considered as regular nodes. This allows a clear analysis of the relationship between each regular node and TNs in the switch matrix.

Let a represent the number of TNs in the same row as (m,n), in which case node (m,n) will be utilized by the path corresponding to the TN. Similarly, let b represent the number of TNs on the same column as (m,n), in which case node (m,n) will be utilized by the path corresponding to the TN. For example, in Figure 1, selecting either node (3,3), (3,4), or (3,5) in the third row or selecting either node (1,2) or (2,2) in the second column as the TN will utilize node (3,2). Thus, a and b of node (3,2) are equal to 3 and 2, respectively.

In a crossbar-based RF MEMS switch matrix including an SR-Crossbar one, where ports are conventionally configured, without losing generality, one can assume that the input ports are on the left side and output ports are on the bottom side. Therefore, it can be derived that

(1) a=N−n, b=m−1,

Here, we consider the case where the number of input and output ports in the switch matrix are equal, i.e., M=N, which is the case for most non-blocking switch matrices.

For a fully configured switch matrix, there are a total of M TNs, with each TN determining one signal path. Based on the non-blocking nature of the crossbar topologies, it is known that within the same row and column of the matrix, there is only one TN. Therefore, the interconnectivity between the ports of the switch matrix can be uniquely determined by the positions of all the TNs. Thus, analyzing all possible interconnections between the input and output ports can be simplified to analyzing the TN positions of all the paths. In one single RF signal path, there is only one TN, and the other nodes are considered as normal nodes. As a result, different distributions of TNs correspond to different utilization scenarios for the nodes on the paths.

Hence, generating a specific port configuration can be simplified to choose M TNs locations according to the requirements and within the regulation of the non-block crossbar topology.

For node (m,n), the probability of its utilization can be divided into four parts:

1. The TNs that will utilize node (m,n) only exist in the same row, of which the probability is calculated as follows:
  (2) $$\begin{array}{cc} P_{1} & =a\times \frac{\left(M-2\right)!}{\left(M-2-b\right)!}\times \left(M-1-b\right)!\times {\left(M!\right)}^{-1}\\ & =a\times \left(M-2\right)!\times \left(M-1-b\right)\times {\left(M!\right)}^{-1} \end{array}$$
  Here, let a be Factor One, (M−2)!/(M−2−b)! be Factor Two, (M−1−b)! be Factor Three, and ${\left(M!\right)}^{-1}$ be Factor Four. The probability can be deduced by analyzing the locations of M TNs. The analysis and calculation process for $P_{1}$ is as follows:
  (1) First, select a TNs in the same row that will utilize node (m,n) as the TN. This gives Factor One.
  (2) Second, start from the first row, one by one until the b-th row, to choose the TNs. Since the TN utilizing node (m,n) can only be in the n-th row with node (m,n), the nodes in column n cannot be selected as the TNs in this step. The total number of TN selection schemes is (M−2)!/(M−2−b)!, resulting in Factor Two.
  (3) Third, starting from the (b+1)-th row until the M-th row, choose the TNs. The first two steps have already completed the selection of b+1 TNs. The remaining (M−1−b) TNs have a total selection scheme of (M−1−b)!, resulting in Factor Three.
  (4) Finally, the total number of random TN selection schemes for the non-blocking interconnection scheme is M!. This serves as the denominator in Factor Four.
2. The TNs which will utilize node (m,n) only exist in the same column, of which the probability is calculated as follows:
  Similar to case above, and given that M=N, we have
  (3) $$P_{2}=b\times \left(M-2\right)!\times \left(M-1-a\right)\times {\left(M!\right)}^{-1},$$
3. The TNs which will utilize node (m,n) exist in the same row and same column at the same time, of which the probability is calculated as follows:
  (4) $$P_{3}=a\times b\times \left(M-2\right)!\times {\left(M!\right)}^{-1},$$
  Similarly, the next steps are as follows:
  (1) First, select a TNs in the same row that will utilize node (m,n) as the TN. This gives Factor One.
  (2) Second, select b TNs in the same column that will utilize node (m,n) as the TN. This gives Factor Two.
  (3) The remaining (M−2) TNs have a total selection scheme number of (M−2)!. This gives Factor Three.
  (4) The total number of TN selection schemes is M!. This serves as the denominator of Factor Four.
4. Node (m,n) is the TN, and the probability is calculated as follows:
  (5) $$P_{4}=1\times \left(M-1\right)!\times {\left(M!\right)}^{-1},$$
  (1) First, select node (m,n) itself as the first TN, and thus the number of selections is 1. This gives Factor One.
  (2) The remaining (M−1) TNs have a total selection scheme of (M−1)!. This gives Factor Two.
  (3) The total number of TN selection schemes is M!. This serves as the denominator of Factor Three.

Therefore, the total probability is as follows:

(6) $$\begin{array}{cc} P\left(m,n\right) & =P_{1}+P_{2}+P_{3}+P_{4}\\ & =\frac{\left(M-1\right)\left(a+b+1\right)-ab}{M\left(M-1\right)}\\ & =\frac{a+b+1}{M}-\frac{ab}{M\left(M-1\right)} \end{array}$$

**Figure 2:**
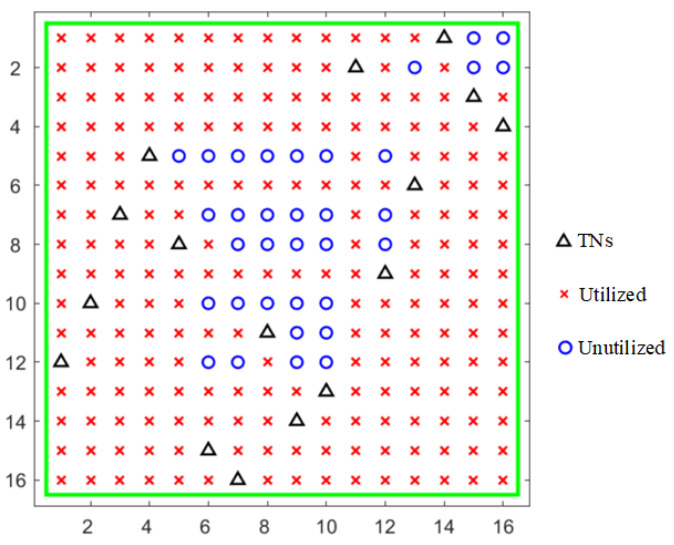
A random port configuration scheme of a 16 × 16 SR-Crossbar switch matrix.

Let us consider the probability distribution of each node in a specific conventional port-configured crossbar matrix. We assume that the number of both the input and output ports is 16, i.e., M=N=16. Here, without a loss of generality, we assume that the inputs are on the left side and the outputs are on the bottom side. Here, we generate a set of port configuration schemes, i.e., a combination of different TN locations in the matrix, and the randomly generated configuration is shown in Figure 2.

As shown in Figure 2, the numbers on the left and bottom are indices of rows and columns, respectively. The input ports are on the left and the output ports are on the bottom. The black triangles represent locations of randomly generated TNs. It can also be observed that there is one and only one TN in the same row or column. The corresponding RF path starts from the left signal input, turns after passing through the TN, and outputs to the output port below it. The red crosses indicate the nodes in the matrix that are utilized in this configuration. The blue circles denote the nodes that are currently unutilized, which are redundant ones. In different configuration schemes, the locations of TNs vary, leading to differences in the occupied and redundant nodes within the matrix.

**Figure 3:**
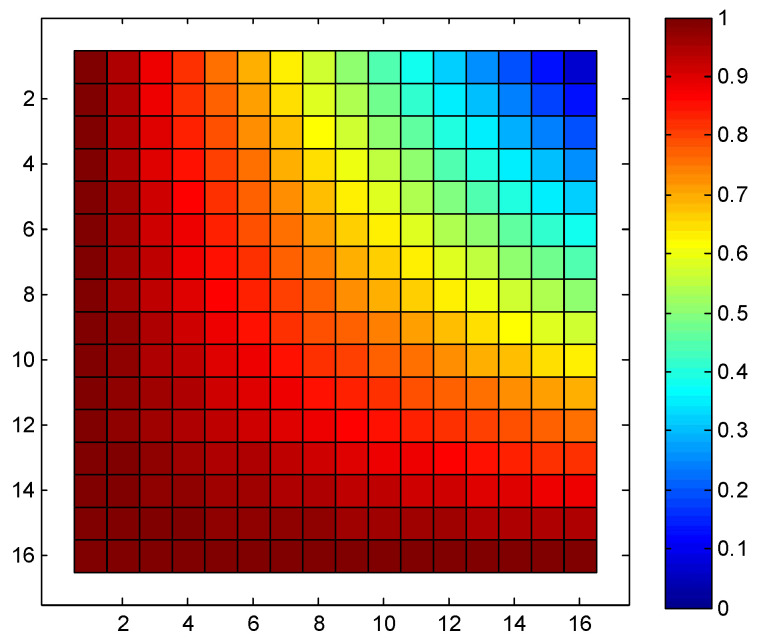
Node utilization probability of a 16 × 16 SR-Crossbar switch matrix.

Based on Equation (6), we can derive the probability of each node’s utilization in a 16 × 16 SR-Crossbar RF MEMS switch matrix when any input and output port is connected in a non-blocking manner. The node utilization probability of this is shown in Figure 3.

In the aforementioned switch matrix, the minimum probability of utilization for each node is 0.0625, the maximum is 1, and the standard deviation is 0.2226. The consistency of node utilization probabilities is relatively poor. As previously analyzed, it is evident that nodes closer to the input and output ports have higher utilization probabilities, while those farther away have lower probabilities. We aim to optimize the ports to achieve a more balanced utilization probability for these nodes, thereby enhancing the dynamic consistency of the switch matrix.

Now that we have obtained the probabilities of each node being utilized in the SR-Crossbar switch matrix under the random non-blocking interconnection of ports, in order to achieve the optimal dynamic consistency of the matrix, we need to make the probability of utilization for each node vary as little as possible.

## 3. Optimization

Based on the basic characteristics of SR-Crossbar topology, we conducted an analysis to obtain the utilization probabilities of each node in the crossbar switch matrix, and its utilization probabilities of nodes follow Equation (6). However, the values of parameters a and b for different input and output port configurations are different. Therefore, determining the port configuration scheme that leads to higher consistency in utilization probabilities for each node in the matrix can be simplified to finding the values of a and b corresponding to different port configuration schemes.

Due to the fact that the SR-Crossbar topology is composed from the basic building blocks through sequential rotation, the SR-Crossbar RF MEMS switch matrix possesses rotational symmetry. Therefore, without a loss of generality, we can assume that all input ports are on the rows of the switch matrix, and all output ports are on the columns. Furthermore, thanks to the rotational symmetry, the nodes in the SR-Crossbar matrix can route signals according to the designated operating mode, irrespective of the side where the input or output ports are positioned. This implies that inputs or outputs in an SR-Crossbar are not limited to being on one side. Thus, determining the entire port configuration scheme for the switch matrix can be simplified to deciding whether each row’s input ports are on the left or right side and whether each column’s output ports are on the top or bottom side.

Meanwhile, regardless of the port configuration scheme, for a non-blocking SR-Crossbar switch matrix with equal numbers of input and output ports, there are a total of M(M=N) turning nodes (TNs). Each TN determines one input–output RF signal path. Additionally, according to the characteristics of a non-blocking topology, each row and each column in the matrix has exactly one and only one TN. In this scenario, for each path in the switch matrix, there are four possible directions of signal flow: left–down (LD), right–down (RD), left–up (LU), and right–up (RU).

Therefore, the expressions for a and b in Formula 6 for the utilization probability of each node are as follows:

(7) $$a=\left\{\begin{array}{c} N-n,\;\;\;\;\mathrm{Type}\;\mathrm{LD}\\ n-1,\;\;\;\;\;\mathrm{Type}\;\mathrm{RD}\\ N-n,\;\;\;\;\mathrm{Type}\;\mathrm{LU}\\ n-1,\;\;\;\;\;\mathrm{Type}\;\mathrm{RU} \end{array}\right.,\;b=\left\{\begin{array}{c} m-1,\;\;\;\;\mathrm{Type}\;\mathrm{LD}\\ m-1,\;\;\;\;\;\mathrm{Type}\;\mathrm{RD}\\ M-m,\;\;\;\mathrm{Type}\;\mathrm{LU}\\ M-m,\;\;\;\;\mathrm{Type}\;\mathrm{RU} \end{array}\right.,$$

The key to establishing the optimization model is obtaining the probability function for the utilization of each node in the matrix. However, discussing the values of a and b in different cases makes it difficult to establish a simplified and analytical function.

Therefore, we rewrite the above equation to

(8) $$a={\left(-1\right)}^{x_{m}}\cdot n-{\left(-N\right)}^{x_{m}},b={\left(-1\right)}^{y_{n}}\cdot m-{\left(-M\right)}^{y_{n}},$$

Here, $x_{m}$ and $y_{n}$ can take values of 0 or 1, where $x_{m}=0$ or 1 represents the input port on the right or left side, respectively, and $y_{n}=0$ or 1 represents the output port on the bottom or top side, respectively. Equation (1) in the previous text corresponds to a specific case where $x_{m}=1$ and $y_{n}=0$.

In order to achieve the highest consistency in utilization probabilities for each node, we aim to find a set of values for $x_{m}$ and $y_{n}$ that minimizes the standard deviation of P(m,n); hence, we have the objective function, which is min{σ}. Here,

(9) $$\sigma =\sqrt{\frac{1}{MN}\sum_{m,n}{\left(P\left(m,n\right)-\mu \right)}^{2}},$$

In which

(10) $$\mu =\frac{1}{MN}\sum_{m,n}P\left(m,n\right),$$

Here, we have established the port configuration optimization model along with its objective function. For a specific switch matrix, we simply need to substitute the given values of the switch ports, i.e., the values of M and N as mentioned in the previous text, into Equations (6), (8)–(10) to obtain the optimal solution.

For example, in the case of a 16 × 16 matrix, which means M=N=16, and utilizing a PSO (Particle Swarm Optimization) algorithm to solve for min{σ}, we obtain the optimal solution as

$$X=\left[x_{m}\right]=\left[1\;1\;1\;1\;1\;1\;1\;1\;0\;0\;0\;0\;0\;0\;0\;0\right]\;Y=\left[y_{n}\right]=\left[0\;0\;0\;0\;0\;0\;0\;0\;1\;1\;1\;1\;1\;1\;1\;1\right]$$

**Figure 4:**
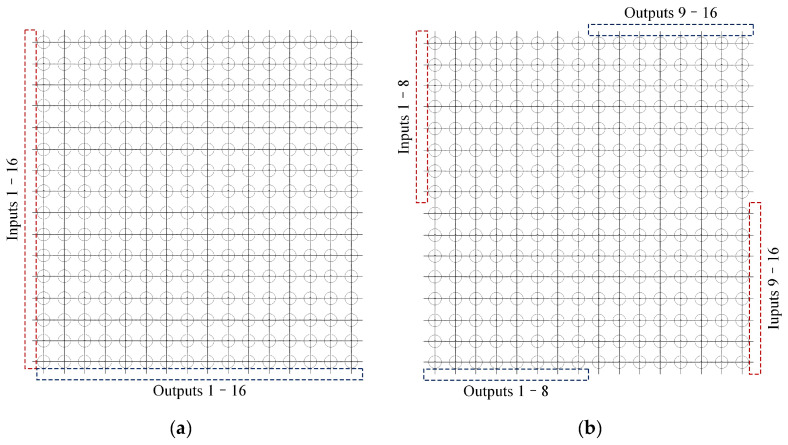
Port configuration scheme of a 16 × 16 SR-Crossbar switch matrix: (**a**) conventional port configuration; (**b**) optimized port configuration.

At this point, the simplified schematic diagram of the switch matrix port configuration is as shown in Figure 4. Figure 4a indicates the conventional port configuration of a 16 × 16 SR-Crossbar switch matrix, while Figure 4b illustrates an optimized version of it. In a conventional port configuration, the inputs or outputs are on the same side of the matrix. Thanks to the rotational symmetric nature of the SR-Crossbar topology, the port configuration scheme can be optimized by adjusting positions. In an optimized port configuration, half of the inputs or outputs are on the same side, while the other half are on the opposite side.

**Figure 5:**
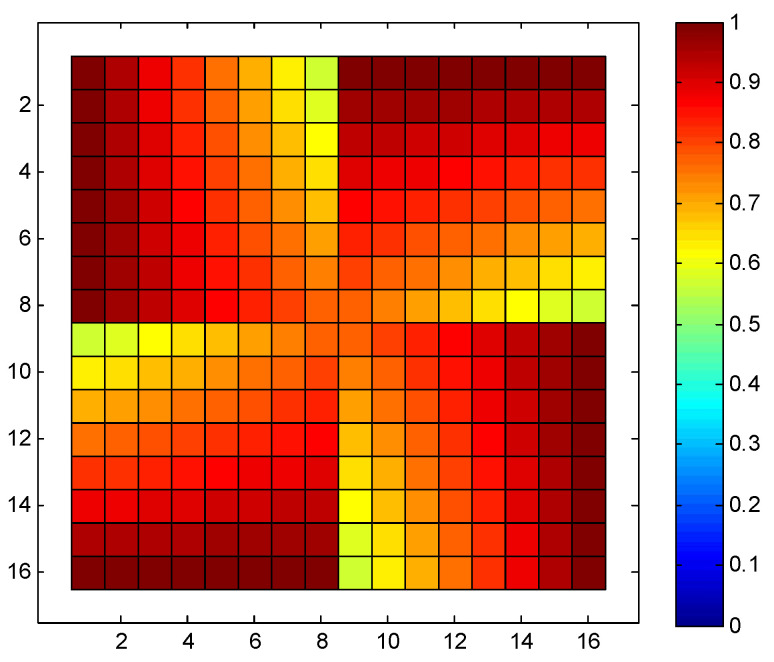
Optimized node utilization probability of a 16 × 16 SR-Crossbar switch matrix.

The probability distribution of each node in the optimized matrix is shown in Figure 5. It is evident from the figure that the utilization probability distribution of each node in the entire switch matrix is more uniform compared to the conventional port configuration scheme in Figure 3. The minimum and maximum probabilities of node utilization are 0.5625 and 1, respectively, with a standard deviation of 0.1171. The standard deviation has significantly reduced compared to the original matrix. As a result, the overall utilization probability of nodes in the switch matrix is more balanced, leading to a significant improvement in the dynamic consistency of the entire switch matrix.

The previous analysis focused on an SR-Crossbar RF MEMS switch matrix, obtaining the utilization probability for each node. Meanwhile, a detailed port configuration optimization was conducted for the SR-Crossbar structure. An optimization model was established, providing an objective function to maximize the consistency of node utilization probability. A specific switch matrix, a 16-input 16-output SR-Crossbar RF MEMS switch matrix, was compared between the conventional port configuration scheme and the optimized port configuration scheme. The results showed that the optimized port configuration scheme reduced the standard deviation of the node utilization probability by 47.4% compared to the conventional scheme, greatly improving the dynamic consistency of the SR-Crossbar-type RF MEMS switch matrix.

As analyzed earlier, the consistency of the switch matrix includes both dynamic and static aspects. Optimizing the consistency of RF signal path lengths is one of the most effective methods for static consistency.

Similar to the previous method, we aim to find an optimal port configuration scheme that minimizes the differences in lengths of each path in the SR-Crossbar switch matrix.

We employ a similar analytical process as described above. However, in contrast to the utilization probability for each node discussed earlier, our focus here is on the length of the RF signal paths, which refers to the number of nodes utilized in each path.

The length of the path with node (m,n) as the turning point TN is denoted as L(m,n), and can be expressed as

(11) $$L\left(m,n\right)=\left(N-a\right)+\left(M-b\right)-1,$$

where a and b are defined the same as in Equation (8). Similarly, we aim to minimize the standard deviation of L(m,n) for all combinations of m and n, with the objective function min{σ’}.

(12) $$\sigma^{\prime }=\sqrt{\frac{1}{MN}\sum_{m,n}{\left(L\left(m,n\right)-\mu^{\prime }\right)}^{2}},$$

In which

(13) $$\mu^{\prime }=\frac{1}{MN}\sum_{m,n}L\left(m,n\right),$$

The obtained set of $x_{m}$ and $y_{n}$ is interesting. The optimized solution, with the objective of minimizing the probability standard deviation, is the same as it is in the former text:

$$X=\left[x_{m}\right]=\left[1\;1\;1\;1\;1\;1\;1\;1\;0\;0\;0\;0\;0\;0\;0\;0\right]\;Y=\left[y_{n}\right]=\left[0\;0\;0\;0\;0\;0\;0\;0\;1\;1\;1\;1\;1\;1\;1\;1\right]$$

**Figure 6:**
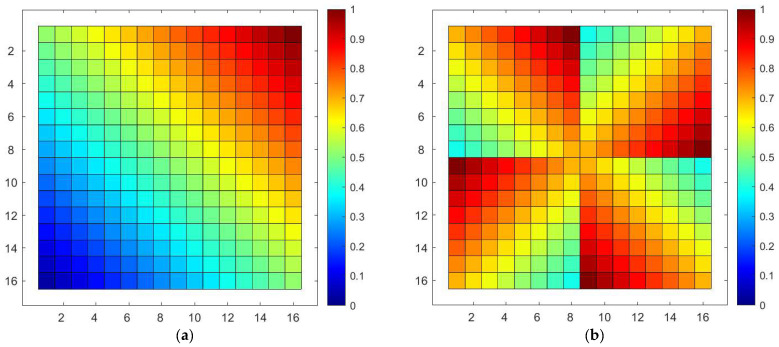
Normalized path lengths before and after port configuration optimization of SR-Crossbar switch matrix: (**a**) normalized path lengths in conventional port configuration of SR-Crossbar switch matrix; (**b**) normalized path lengths in optimized port configuration of SR-Crossbar switch matrix.

For better visualization, we normalize the path lengths by dividing each path length by the maximum value among them all. The normalized path lengths in conventional and optimized port configuration SR-Crossbar switch matrices are shown in Figure 6a and Figure 6b, respectively.

In the conventional port configuration scheme, the minimum and maximum lengths of each path in the matrix are 1 and 31, respectively. The standard deviation is 6.5320. In the optimized scheme, the minimum and maximum lengths of each path are 9 and 23, respectively, with a standard deviation of 3.2467. The standard deviation is reduced by 50.3% compared to the conventional port configuration scheme.

**Figure 7:**
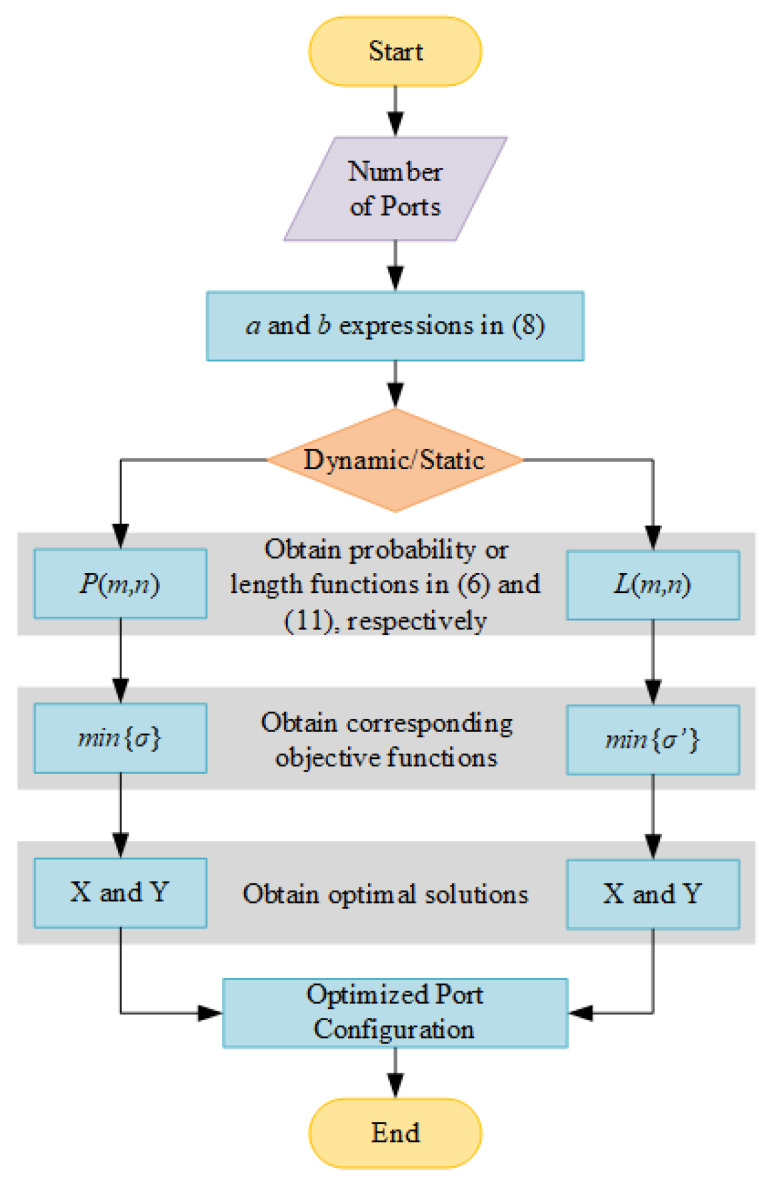
Flowchart of obtaining the optimized port configuration.

For a given size of the SR-Crossbar switch matrix, the process of obtaining the optimized port configuration is shown in Figure 7. The steps are as follows:

1. Input the number of ports of the switch matrix, denoted as M and N in this paper. For non-blocking switch matrices, typically M=N.
2. Substitute M or N into Equation (8) to obtain expressions for a and b.
3. Determine whether the optimization objective is dynamic or static consistency.
4. Obtain the utilization probability function of switch units or the path length function, as shown in Equations (6) and (11), respectively. These functions correspond to the optimization of dynamic and static consistency, respectively.
5. Formulate the corresponding objective functions, where σ and σ’ are expressed in Equations (9) and (12).
6. Solve the objective functions to obtain X and Y.
7. Based on the X and Y obtained, determine the optimized input and output port configuration scheme for the switch matrix.

Multiple optimized port configuration schemes for SR-Crossbars with different input and output numbers are calculated. For the same matrix, whether aiming for the best consistency in node utilization probability or path length, the results of the optimization are the same. Therefore, for these SR-Crossbar RF MEMS switch matrices, the optimal port configuration scheme calculated in the manner proposed in this article will significantly enhance both the dynamic and static consistency of the matrix. This provides two benefits for the switch matrix: better consistency in the performance degradation of each switch unit, which maximizes the lifespan and stability; and for each conducting path in the switch matrix, the RF performance varies less, resulting in better channel consistency. In practical usage scenarios, the number of ports in the matrix is not expected to be excessively large. We performed calculations for matrices with up to 1000 ports using the optimization scheme proposed, and the results obtained all conform to the pattern where half of the ports continuously distributed on one side and the other half on the opposite side.

The optimization above demonstrated the case of a 16×16 switch matrix for simplicity. In reality, when the dimensions of the switch matrix are higher, improving its consistency becomes even more important. The proposed port optimization scheme in this article can optimize the lifespan and overall performance of the switch matrix by simply optimizing the port positions, while keeping the topology of the switch matrix and the switch units unchanged. Hence, the proposed optimization has significant practical value and high feasibility.

## 4. Verifications

To validate the proposed port configuration optimization scheme, we conducted simulations on a specific metal-contact serial RF MEMS switch matrix. The optimized 16 × 16 SR-Crossbar RF MEMS switch matrix achieves optimal dynamic and static consistency, as discussed above. For metal-contact RF MEMS switch matrices, dynamic consistency primarily manifests in the variation in contact resistance [15]. While static consistency is mainly reflected in the consistency of path lengths in this article, in actual RF MEMS switch matrices, it is mainly reflected in differences in path losses. Therefore, we conducted detailed simulations on an actual SR-Crossbar switch matrix composed of metal-contact RF MEMS switch units to study the variations in contact resistance and path losses. Considering the model complexity in a full-wave simulation using the Finite Element Method (FEM), we chose an 8×8 SR-Crossbar RF MEMS switch matrix as the simulation object to improve simulation accuracy, since the model proposed in the article applies to any given matrix dimension. Appling M=N=8 to the equations above, the optimized port configuration for this matrix will be X=[1 1 1 1 0 0 0 0] and Y=[0 0 0 0 1 1 1 1], and the corresponding switch unit utilization probability standard deviations with the optimized and conventional port configuration are 0.12 and 0.22, respectively, and for path lengths, they are 1.59 and 3.27.

**Figure 8:**
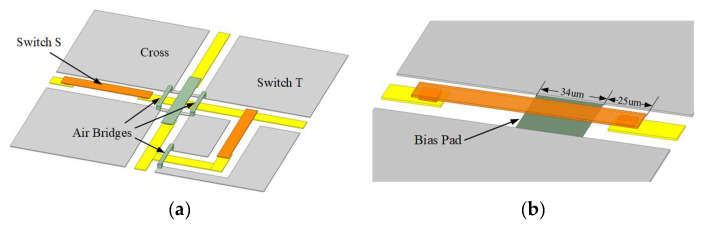
Simulated 8×8 SR-Crossbar switch: (**a**) a building block with switch units; (**b**) cantilever switch with bias pad indicated.

**Figure 9:**
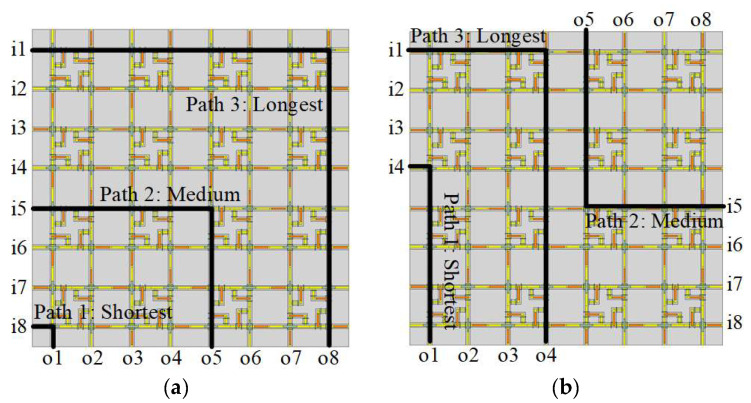
Simulated 8×8 SR-Crossbar switch matrix: (**a**) conventional port configuration; (**b**) optimized port configuration.

**Figure 10:**
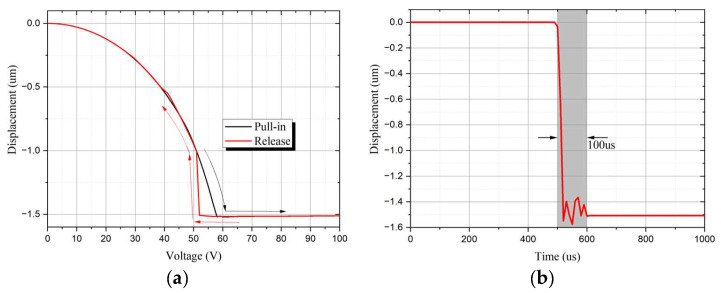
Simulated SR-Crossbar switch unit: (**a**) pull-in and release voltage with arrows indicating pull-in or release direction; (**b**) switching time.

The same cantilever RF MEMS switch unit as that proposed in article [14], which introduced the SR-Crossbar topology, was adopted in the simulation. The length of the switch cantilever beam is 115 µm, the width is 15 µm, and the height is 2.5 µm, with an air gap of 1.5 µm between the contact points. The theoretical capacitance value in the OFF state is 1.44 pF, and the simulated total capacitance value is 1.91 pF with a fringing effect. To suppress unexpected modes such as microstrip mode and slot mode, air bridges are introduced with a height of 2.5 µm and width of 5 µm. The simulated switch matrix model is shown in Figure 8 and Figure 9. In Figure 8, the cantilever RF MEMS switch unit and the building block is illustrated. Appling sufficient voltage to the bias pad, the cantilever will be pull-in, and the corresponding RF path is switched on. The simulated pull-in and release voltage is shown in Figure 10a, and switch time is shown in Figure 10b. It can be seen that the switch has a pull-in and release voltage of 58 V and 52 V, respectively. In Figure 10b, a sufficient voltage of 60 V is applied to the bias pad at the time of 500 μs, and the switch stays at a stable pull-in state at 600 μs. Thus, it has a switching time of 100 μs.

**Figure 11:**
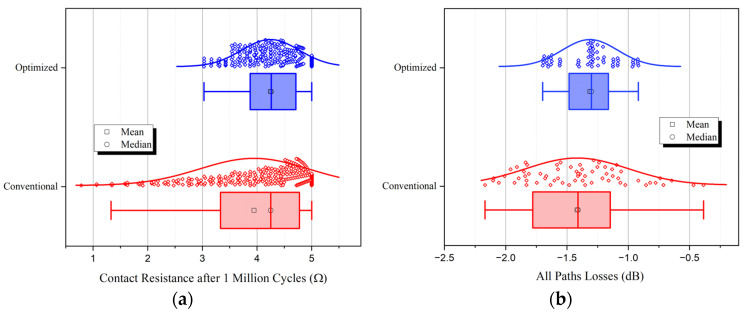
Simulated results of SR-Crossbar switch matrix with optimized (blue) and conventional (red) port configurations: (**a**) contact resistance of switch units in the matrix after 1 million cycles; (**b**) path losses of all possible non-blocking paths in an 8×8 SR-Crossbar switch matrix.

For working metal-contact RF MEMS switches, the contact resistance is typically less than 1 ohm, with a typical value of 0.5 ohm. Additionally, the contact resistance increases with the utilization of the switches. The existing simulation tools have difficulty accurately estimating the contact loss of RF MEMS switches, along with their corresponding changes in contact resistance. According to references [9,15], before switch failure, the contact resistance linearly increases versus the number of switch utilizations, reaching 5 ohms after one million switch cycles. Therefore, in the simulation we assume that the change in contact resistance versus switch cycles complies with

$$R_{c}=0.5+4.5\times 10^{-6}\times n_{c}$$

where $R_{c}$ and $n_{c}$ represent contact resistance and switch cycles, respectively. One million simulation experiments were conducted, and for each experiment, random non-blocking connections were made between the input and output ports of the matrix. The number of times each switch was utilized was recorded, which was used to calculate the resistance based on the function given above. For all 128 units in the matrix, the simulated contact resistance data distribution after 1 million cycles is shown in Figure 11a.

Furthermore, we conducted a full-wave FEM simulation from 0 to 40 GHz on the path losses in an 8×8 SR-Crossbar RF MEMS switch matrix before and after port optimization. Data were collected at the central frequency of 20 GHz for comparison. The conventional and optimized simulation model of the 8×8 SR-Crossbar switch matrix is shown in Figure 9, with a dimension of 1.7×1.7 mm^2^. All 64 different path losses were studied to verify the static consistency of losses. The simulation results are shown in Figure 11b.

All data samples in Figure 11 are one-dimensional samples, and the scatter data points in the figure are all jittered for the better visualization of data distribution. Curves in the figure are normal distribution curves of the samples. The blue and red plots represent the simulation results of the optimized port configuration scheme proposed in this article and the conventional one, respectively. Compared to the red plot, the box lengths in the blue boxplot are shorter, with fewer outliers, and the scatter of data points is more concentrated. These indicate that the parameters of the matrix using the optimized port configuration scheme are more consistent. Specifically, after one million cycles, the standard deviations of contact resistance with conventional and optimized port configuration are 1.00 and 0.51, respectively. The standard deviations of path losses with the conventional and optimized port configuration are 0.42 and 0.23, respectively. The standard deviations of these two criteria are approximately halved after optimization, which agrees very well with those calculated theoretically using the model proposed in the article.

**Figure 12:**
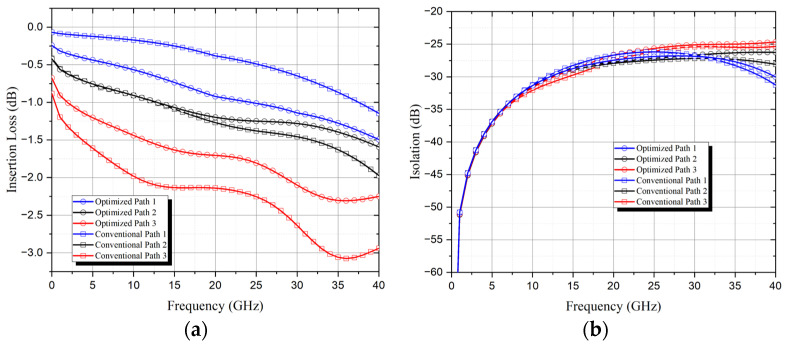
Simulated 8×8 SR-Crossbar switch matrix with optimized and conventional port configurations: (**a**) insertion losses of Path 1, 2 and 3; (**b**) isolations of Path 1, 2 and 3.

Three typical paths, shortest, medium, and longest, are illustrated in Figure 9 to showcase the RF performance across 0–40 GHz for the matrices. The simulated results are presented in Figure 12, indicating that both matrices exhibit good isolation, maintaining better than 25 dB for all three paths within the 0–40 GHz range. However, differences arise in insertion loss: for each frequency, the gap between insertion loss of the longest and shortest paths in the optimized switch matrix remains approximately half that of a conventional matrix. The largest insertion loss gap in the optimized matrix is 1.05 dB at 33 GHz, whereas it reaches 2.20 dB at 34 GHz in the conventional counterpart.

Compared with the L-shaped matrices proposed in [13], this study achieves similar performance in enhancing matrix static consistency, thus reducing the performance gap between the best and worst paths. However, the SR-Crossbar topology in this study demonstrates superior compactness with a 1.7×1.7 mm^2^ 8×8 matrix compared to a 4×4 mm^2^ 4×4 matrix. Notably, our proposed optimization scheme achieves the best dynamic consistency among all related works for the first time.

## 5. Conclusions

The performance consistency of RF MEMS switch matrices is an important indicator. This article proposes the concepts of dynamic and static consistency of the matrix. Starting from optimizing the dynamic consistency of the RF MEMS switch matrix with an SR-Crossbar structure, the article conducts detailed research on its port configuration scheme. The utilization probability for each node in the matrix and the optimization objective function are given to achieve the best consistency of node utilization probability. By using the method proposed in the article, the best port configuration scheme for a specific 16×16 matrix is obtained. Compared to the conventional port configuration scheme, the standard deviation of node utilization probability is reduced by 47.4%, significantly optimizing the dynamic consistency of the matrix. Furthermore, this port configuration scheme also applies to achieving the highest static consistency, with which RF signal path variation is minimized. The standard deviation of path lengths is reduced by 50.3% compared to the conventional port configuration scheme, and thus static consistency is also significantly optimized. Simulations to demonstrate the optimized dynamic and static consistencies were also conducted by analyzing the switch unit contact resistance and matrix path losses, and the simulated results agreed very well with the theoretical ones calculated using the model proposed. 

Essentially, the port configuration optimization proposed in this paper is an enhanced topology based on the SR-Crossbar. Its significance lies in providing researchers with a reference during the design phase of RF-MEMS switch matrices. By solely adjusting port positions in the topology without affecting other designs, researchers can achieve improvements in both the static and dynamic consistency of the matrix. Additional performance optimizations are still possible by separately adjusting switch unit and interconnection designs, and this introduces additional degrees of freedom for optimizing matrix consistency and indicates evident universality and operability of the proposed port configuration scheme. Moreover, the proposed topology is based on the SR-Crossbar, a widely used crossbar variant in the RF switch industries. With no other adjustments introduced apart from port positions, this topology can be widely applied to various applications with no specific hardware or software requirements.

Currently, the optimization of port positions is limited to SR-Crossbar due to its rotational symmetry. Future research could explore the operational modes of nodes in different topologies when facing various signal input and output directions, thereby initiating optimization work on port position configurations. Additionally, future research endeavors could focus on providing purely mathematical proofs of conjectures regarding the generalization of the optimized port configuration patterns as discussed above, where half of the ports are continuously distributed on one side and the other half on the opposite side, possessing 90° rotational symmetry at the same time.

## Data Availability

The data used to support the finds of this research are available from the corresponding author upon request.

## References

[1] Iannacci J. (2015). RF-MEMS: An enabling technology for modern wireless systems bearing a market potential still not fully displayed. Microsyst. Technol..

[2] Papaioannou G., Exarchos M.-N., Theonas V., Wang G., Papapolymerou J. (2005). Temperature study of the dielectric polarization effects of capacitive RF MEMS switches. IEEE Trans. Microw. Theory Tech..

[3] Ma L., Soin N., Daut M.H.M., Hatta S.F.W.M. (2019). Comprehensive Study on RF-MEMS Switches Used for 5G Scenario. IEEE Access.

[4] Iannacci J. (2022). Expectations versus actual market of RF-MEMS: Analysis and explanation of a repeatedly fluctuating scenario. RF-MEMS Technology for High-Performance Passives.

[5] Zorpette G. (2020). RF MEMS deliver the “ideal switch”: After two decades of development, MEMS-based RF switches are finally finding real-world uses. IEEE Spectr..

[6] Zhu H., Cui W., Li Y., Song M. (2023). Design and Analysis of a Fluid-Filled RF MEMS Switch. Sensors.

[7] Basu A., Adams G.G., McGruer N.E. (2016). A review of micro-contact physics, materials, and failure mechanisms in direct-contact RF MEMS switches. J. Micromechanics Microengineering.

[8] Kim C.H. (2012). Mechanically Coupled Low-Voltage Electrostatic Resistive RF Multithrow Switch. IEEE Trans. Ind. Electron..

[9] Liu Y., Bey Y., Liu X. (2016). Extension of the Hot-Switching Reliability of RF-MEMS Switches Using a Series Contact Protection Technique. IEEE Trans. Microw. Theory Tech..

[10] Di Nardo S., Farinelli P., Kim T., Marcelli R., Margesin B., Paola E., Pochesci D., Vietzorreck L., Vitulli F. (2013). Design of RF MEMS based switch matrix for space applications. Adv. Radio Sci..

[11] Fomani A.A., Mansour R.R. (2009). Monolithically Integrated Multiport RF MEMS Switch Matrices. IEEE Trans. Microw. Theory Tech..

[12] Daneshmand M., Mansour R.R. (2011). RF MEMS Satellite Switch Matrices. Microw. Mag. IEEE.

[13] King Yuk C., Daneshmand M., Mansour R.R., Ramer R. (2009). Scalable RF MEMS Switch Matrices: Methodology and Design. IEEE Trans. Microw. Theory Technol..

[14] Zhou W., Sheng W., Cui J., Han Y., Ma X., Zhang R. (2019). SR-Crossbar topology for large-scale RF MEMS switch matrices. IET Microwaves, Antennas & Propagation.

[15] Chow L.W. (2006). Thermal, Residual Stress and Contact Reliability of RF MEMS: Fundamental Physics and Improvements. Ph.D. Theses.

